# Maternal neglect alters reward-anticipatory behavior, social status stability, and reward circuit activation in adult male rats

**DOI:** 10.3389/fnins.2023.1201345

**Published:** 2023-07-14

**Authors:** Ermis Ryakiotakis, Dimitra Fousfouka, Antonios Stamatakis

**Affiliations:** ^1^Laboratory of Biology-Biochemistry, Faculty of Nursing, School of Health Sciences, National and Kapodistrian University of Athens, Athens, Greece; ^2^MSc Program in Molecular Biomedicine, Medical School of National and Kapodistrian University of Athens, Athens, Greece

**Keywords:** maternal neglect, reward anticipation, social competition, vPFC, NAc, pCREB

## Abstract

**Introduction:**

Adverse early life experiences affect neuronal growth and maturation of reward circuits that modify behavior under reward predicting conditions. Previous studies demonstrate that rats undergoing denial of expected reward in the form of maternal contact (DER-animal model of maternal neglect) during early post-natal life developed anhedonia, aggressive play-fight behaviors and aberrant prefrontal cortex structure and neurochemistry. Although many studies revealed social deficiency following early-life stress most reports focus on individual animal tasks. Thus, attention needs to be given on the social effects during group tasks in animals afflicted by early life adversity.

**Methods:**

To investigate the potential impact of the DER experience on the manifestation of behavioral responses induced by natural rewards, we evaluated: 1) naïve adult male sexual preference and performance, and 2) anticipatory behavior during a group 2-phase food anticipation learning task composed of a context-dependent and a cue-dependent learning period.

**Results:**

DER rats efficiently spent time in the vicinity of and initiated sexual intercourse with receptive females suggesting an intact sexual reward motivation and consummation. Interestingly, during the context-dependent phase of food anticipation training DER rats displayed a modified exploratory activity and lower overall reward-context association. Moreover, during the cue-dependent phase DER rats displayed a mild deficit in context-reward association while increased cue-dependent locomotion. Additionally, DER rats displayed unstable food access priority following food presentation. These abnormal behaviours were accompanied by overactivation of the ventral prefrontal cortex and nucleus accumbens, as assessed by pCREB levels.

**Conclusions/discussion:**

Collectively, these data show that the neonatal DER experience resulted in adulthood in altered activation of the reward circuitry, interfered with the normal formation of context-reward associations, and disrupted normal reward access hierarchy formation. These findings provide additional evidence to the deleterious effects of early life adversity on reward system, social hierarchy formation, and brain function.

## 1. Introduction

It is well established that early life experiences are vital for the survival and healthy development of most animals including humans (Pollak et al., 2005). Disruption of the normal parent–child interactions can have long-lasting effects on the quality of life of the outspring (Lai and Huang, 2011). Indeed, early life stress (ELS) in the form of parental neglect/maltreatment/loss/abuse has been linked to a wide range of mental pathologies including disturbed social cognition combined with poor prognosis in psychological disorders (schizophrenia, bipolar disorder, borderline personality disorder, major depression, and/or post-traumatic stress disorder) (Rokita et al., 2018). Institutionalized or neglected children display abnormal behaviors, cognitive dysfunction, and aberrant brain maturation (Bowlby, 1951; Pryce et al., 2005; Gee et al., 2013; Pollak, 2015). Furthermore, many effects of ELS are long-lasting, persisting even in adulthood. Adults exposed to ELS manifest reward system deficiencies such as lower behavioral drive when faced with an unexpected increase in monetary reward (Niwa et al., 2011; Sheridan et al., 2018) as well as attenuated ventral striatum (VS) response during reward reception and formation of positive social relationships (Mehta et al., 2010; Goff et al., 2013; Hanson et al., 2015).

Studies that employ animal models have associated mother–pup separation with adverse effects on stress responsivity, adaptability, cognitive function, and drug abuse (Liu et al., 2000; Levine, 2001; Sánchez et al., 2001; Delavari et al., 2016). More specifically, disrupting dam–pup interactions can alter the development and activity of the mesolimbic and mesocortical dopaminergic systems that drive motivated behavior (Hall et al., 1999; Matthews et al., 2001; Björklund and Dunnett, 2007; Lovic et al., 2013). The effects on reward perception and goal-oriented behaviors are mediated either directly through the downregulation of DA receptor expression or indirectly through the modulation of VTA function via hypothalamic–pituitary–adrenal axis alterations (Ploj and Nylander, 2003; Zhu et al., 2010; Callaghan and Richardson, 2013). These findings highlight the importance of early age for normal brain and behavioral maturation suggesting that ELS poses a strong etiology for the manifestation of psychiatric diseases mainly through the modification of the reward system.

Early life adversity has also been linked with the occurrence of abnormal social behaviors such as increased aggression and lower social responses in humans (Widom and Brzustowicz, 2006; Goff et al., 2013) or reduced competition, increased play-fight, and increased sexual motivation in rodents (Greisen et al., 2005; Veenema et al., 2006; Benner et al., 2014; Haller et al., 2014; Davis et al., 2020). Normally, social interactions facilitate stress reduction (Lemos et al., 2021) and are essential for PFC maturation (Baarendse et al., 2013). Rodents will readily engage in social interactions (Moy et al., 2004) choosing social contact rather than drug consumption (Sampedro-Piquero et al., 2019; Venniro et al., 2021). Disruption of normal social behaviors of ELS individuals can constitute an additional stressful social environment that could escalate psychopathological progression. Indeed, social stress has been correlated with many conditions such as depression, anxiety, sociophobia, or loss of self-esteem (Björkqvist, 2001; Meyer-Lindenberg and Tost, 2012). In rodents, adverse social interactions have been associated with a wide range of depressive-like behaviors (Hollis and Kabbaj, 2014) such as anhedonia and decreased exploratory activity (Rygula et al., 2005) along with reduced behavioral response in high-reward context (Der-Avakian et al., 2017). Collectively, these studies underlie the potential effects of early life stress on the formation of abnormal social interactions leading to greater pathological implications, enhancing stress response and reward system deregulation.

The dopaminergic system is regarded as the central neural sensor of rewarding experiences that drives motivated behavior toward desired goals. The mesolimbic/mesocortical circuit consists predominantly of specialized neurons that synthesize dopamine (DA) and project from the ventral tegmental area (VTA) to the prefrontal cortex (PFC) and nucleus accumbens (NAc) (Jackson et al., 2001; Del Arco and Mora, 2008; Baik, 2013). Notably, DA release has been correlated with the sense of reward anticipation and acquisition (Haber and Knutson, 2010), while drugs such as amphetamine, which prolong DA presence in the synaptic cleft are extremely addictive (Koob, 1992; Wise and Hoffman, 1992; Di Chiara, 1995). DA-DA receptor binding is crucial for the manifestation of reward-driven behaviors (Wise, 1982). In classical Pavlovian conditioning, cue-reward onset induces phasic DA release from VTA. VTA neuron firing rate on NAc predicts the expected value of an upcoming reward (Schultz et al., 1997). For example, increases in phasic DA release promote learning association between a natural reward (unconditioned stimulus) and the environmental cues that predict it (conditioned stimulus) (Palmiter, 2007).

DA-DA receptor binding engages G-coupled proteins that activate a cAMP-dependent pathway leading to long-term effects via gene transcription modulation (Sassone-Corsi, 2012). A major mediator of cAMP signaling is the cAMP response element binding (CREB) protein that is activated through the phosphorylation of serine 133 and acts as a transcriptional factor for many immediate early genes (Alberini, 2009; Dyson and Wright, 2016). pCREB activation has been linked to associative learning or long-term potentiation (Atkins et al., 1998; Cao et al., 2009), e.g., in NAc during a response to natural reward-predicting cues (cue-reward association) (Shiflett et al., 2009) or in PFC during memory consolidation and reward strategy formation (Bartolotti et al., 2016; Verharen et al., 2020). The importance of early life adversity on reward system dysfunction discussed previously poses CREB phosphorylation status in NAc and PFC during reward learning or anticipation conditions, a focal point that could reveal additional links connecting ELS and abnormal reward signaling.

To simulate maternal neglect, a rather common form of ELS in humans, our laboratory has developed an animal model in which rat pups during post-natal days (PNDs) 10–13 are exposed to a *T*-maze, one arm of which leads to the mother-containing cage where contact with the mother is denied (denial of expected reward) (Panagiotaropoulos et al., 2009). The DER experience is a learning task of a relatively mild negative valence, with additional elements of frustration and delayed care receipt, a condition that accurately resembles human neglect (Stamatakis et al., 2013). It is interesting to note that adult male DER animals have a hypofunctioning serotonergic system and display depressive-like attributes such as reduced sucrose preference and social aggression (Diamantopoulou et al., 2012). Furthermore, DER pups exhibited decreased DA levels in the PFC that persist into adulthood (Diamantopoulou et al., 2013). Moreover, adult male DER rats had fewer glutamatergic neurons and lower neuronal density in the medial orbital and infralimbic cortex accompanied by poor performance in attention set-shifting tasks (Stamatakis et al., 2016). These findings suggest that the DER experience can have long-lasting effects on the PFC in general and specifically on its dopaminergic system, possibly affecting its contribution to the reward circuitry function. Although type-1 dopamine receptor (DR1) protein levels in the PFC and nucleus accumbens (NAc) have been reported to remain intact under basal conditions in adult DER rats (Raftogianni et al., 2014), no relevant data are available concerning the signaling status of the mesocortical/mesolimbic system in tasks that promote conditioned reward.

The purpose of this study was to investigate the undocumented impact of the DER experience on naturally evoked motivated behaviors and their underlying reward circuit mediators, namely the vPFC and the NAc. We hypothesized that the DER experience can disrupt normal responses to natural rewards (Berridge and Robinson, 2003). First, we determined the approach behaviors and preference to a sexually receptive female as well as the latency to initiate sexual behavior in an unrestrictive environment. Receptive female pheromones have been shown to activate the mesolimbic DA system, even prior to the expression of copulatory behavior (Malkesman et al., 2010; Frick et al., 2015; Beny-Shefer et al., 2017). These assays provide insight into both motivational and consummatory aspects of naïve male sexual behavior. Second, we investigated learning performance in a Pavlovian cue-reward association task performed by groups of animals. Earlier experiments studying the learning efficiency of single animals indicated that the DER experience does not impact adult learning efficiency in Morris water maze or rule learning (substrate-food reward association) tasks (Diamantopoulou et al., 2011; Stamatakis et al., 2013) under single animal learning conditions. To address the potential social aspect of reward learning, we used grouped food anticipation (FA) trials to assess how the DER experience can act as a modulator of reward cognition during group learning. We measured the horizontal locomotion, rearing activity, reward quadrant preference, and food access priority of food-restricted (FR) animals in an open field arena. General locomotion and place preference are widely used markers to describe conditioned anticipatory behavior robustness and spatial food reward association (Rubinow et al., 2009; Gallardo et al., 2020). Rearing activity is an exploratory activity that is elevated during anticipation (Lucas et al., 1988), and food access priority is an indicator of the stability of hierarchical structure within the groups (Costa et al., 2021). Additionally, we measured CREB phosphorylation (ser 133) following the final day of FA in NAc and vPFC to explore reward circuit activation during reward anticipation.

## 2. Materials and methods

### 2.1. Animals

Wistar rats used in the following experiments were bred in our colony (EL 25 BIObr 43). Animal living conditions follow the standard guidelines for rat housing: three animals were housed together in a Plexiglas cage with a mesh wire top and had *ad libitum* access to water and food (food pellets, Kounker-Keramari Bros. and Co., Athens, Greece) in a 12 h light/day cycle (07:00 a.m.−19:00 p.m.) at 22–23°C. The bedding (wood chip) was replaced weekly. Prior to birth, each litter was randomly allocated to the control group or the denial of expected reward (DER) group (see below). Litters having 3–6 males and 6–10 animals in total were used in order to have comparable levels of nest conditions and avoid extreme variations in pup body weight and maternal behavior. To avoid disturbing the litter or the mother, cages were not cleaned, but instead wood chip was added. After weaning (PND 22), all animals were left undisturbed (except for weekly cage cleaning) until the start of the behavioral experiments (PND 80).

At weaning, rats were separated from their mothers and housed in the following groups: 3xDER male (DER group) and 3xCTR male (CTR group). Each group was, randomly, assigned to the basal or experimental conditions, and all animals in each cage were littermates: Under **basal conditions**, animals were not behaviorally tested in adulthood, while under experimental conditions, animals were behaviorally tested in adulthood.

All experiments were carried out in agreement with the ethical recommendations of the European Communities Council Directive of 22 September 2010 (2010/63/EU) and approved by our institution's ethical committee (authorization number #291, Athens, 2019). All efforts were made to minimize animal suffering and to reduce the number of animals used.

### 2.2. Neonatal *T*-maze training

A model of mild early life adversity has been developed in our lab, in which pups are trained in a *T*-maze while being denied the expected reward of maternal contact (DER). As previously described in detail (Panagiotaropoulos et al., 2009; Diamantopoulou et al., 2011), we used a custom-made *T*-maze where the cage with the mother and litter was placed at the end of one arm of a *T*-maze, and a cage with a virgin adult female rat was placed at the end of the other arm. Plastic mesh at the gate of each cage prevented physical contact with the animals in either cage. Each rat pup was placed at the center of the *T*-maze, free to explore for 60 secs. If the pup successfully reached the mother-containing cage, it remained in front of the entrance for 20 seconds, and then was returned to the center of the *T*-maze; otherwise, it was gently pushed to the entrance of the mother-containing cage and left there for 20 seconds before returning to the center of the *T*-maze. This was repeated 10 times per day for 4 days from post-natal days 10 to 13 (PND 10–13). Between pups, the *T*-maze was cleaned thoroughly with 70% ethanol. To retain the original home cage smell and amplify the olfactory cues, each day some bedding (one handful) was transferred from the home cage to the mother-containing cage in the *T*-maze. CTR animals did not undergo any training and were left undisturbed with their mother until weaning apart from weekly husbandry procedures.

### 2.3. Behavioral testing

**Figure 1:**
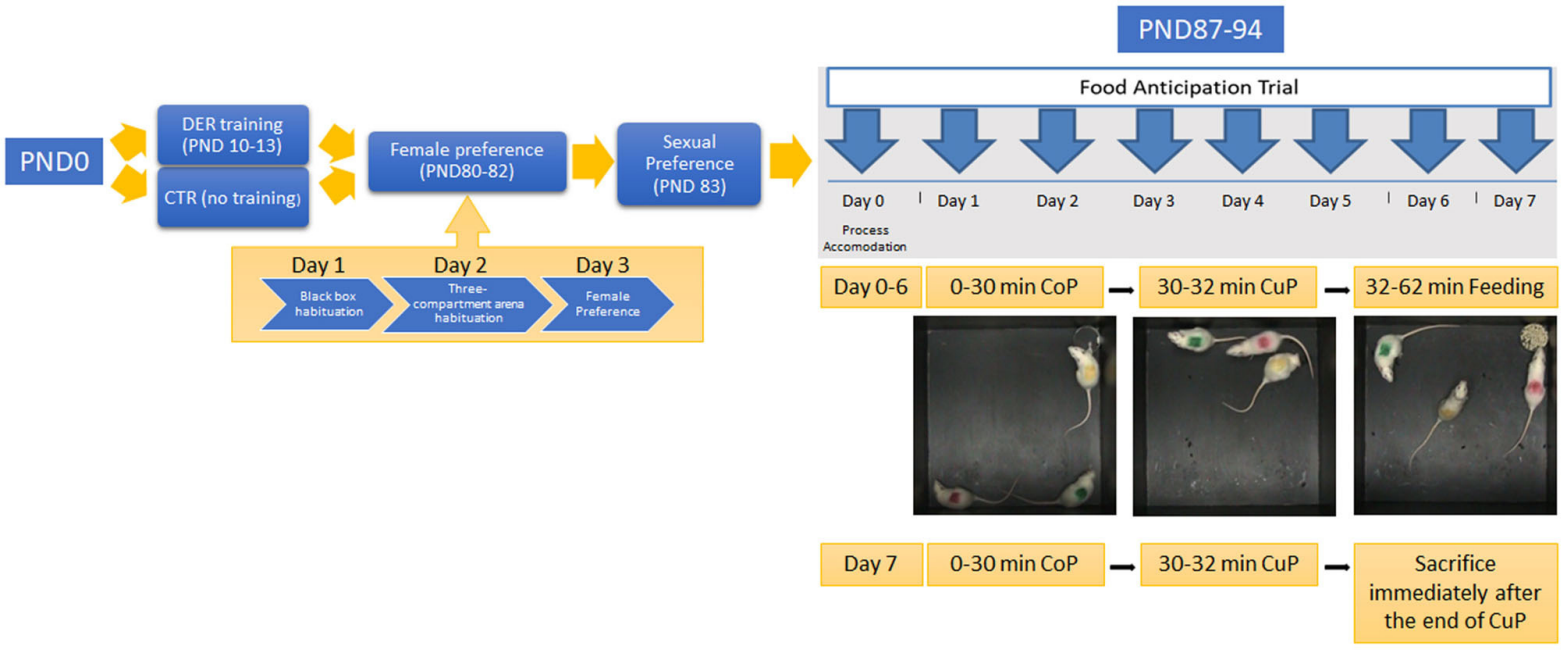
Experimental timeline: the timeline of each experimental procedure. PND 10–13: rat pups of the DER group undergone DER training. Animals of the CTR group remained undisturbed. PND 80–82: adult animals of both groups undergone the female preference test. On PND 80, rats were habituated to the black box arena; on PND 81, they were habituated to the three-compartment arena; and on PND 82, female preference test was conducted. PND 83: sexual behavior was tested. PND 87–94: food anticipation training was carried out for eight consecutive days. Day 0 served as the accommodation trial. Food was removed from the home cage for the rest of FA training. PND 94: animals were killed immediately after the end of day 7 FA training CuP, and brain tissues were harvested.

Animals underwent a series of behavioral testing summarized in Figure 1.

#### 2.3.1. Female preference test

To determine the sexual drive of male rats we utilized: (1) a preference test for a cage containing an unfamiliar, intact, sexually receptive female and (2) sexual behavior of sexually naive males. On the first day of the test (PND 80), each rat was left to move freely in an open field arena (square wooden box with a black painted surface, 57 × 57 × 57 cm with an open top) for 5 min to habituate with the experimental space (black box habituation). On the second day (PND 81), the same open field arena was divided into three compartments with Plexiglas: two equal ones (left and right, 41.5 × 28 cm each) and a connecting corridor (15.5 × 57 cm). At the center of each of the compartments, a small metal-wired cage (18.5 × 13 × 8.5 cm height) was placed. Each rat was placed at the center of the passage connecting the two larger compartments, facing toward either the right or left compartment (equally balanced animals), and left free to explore the setup for 10 min (three-compartment arena habituation). On the third day (PND 82), we used the previous setup with the addition of a sexually receptive female rat in one cage and a rat-like cotton dummy in the other. Again, each experimental rat was placed at the center of the connecting passage, facing either toward the female or the dummy (equally balanced animals), and left free to explore the setup for 10 min (female-containing cage preference trial). On all days, between different animals, the arena was thoroughly cleaned with 70% ethanol.

Receptivity of the females was confirmed by the microscopical observation of vaginal smear, immediately before the initiation of female preference tests or sexual preference trials (see Section 2.3.2). Smears containing large non-nucleated cornified cells indicated oestrus and thus sexual receptivity. In addition, the receptivity of each female was verified by exposure to an experienced male and registration of ear wiggling and dart behavior by the female. In total, 20 female animals were used.

Behavior on all days was recorded using a digital video camera. Auto exposure and autofocus features were disabled, and zoom was manually fixed. The EthoVision 7XT (Noldus, Netherlands) software was used, employing the static subtraction method to track animals during black box as well as during female preference tests. In the black box habituation analysis, the floor surface of the wooden box was designated as the arena, and the central area of the surface (18.8 × 18.8 cm) was designated as the center zone. In female preference analysis, the arena was divided into the corridor, “female,” and “dummy” zones. EthoVision calculated the total distance moved, entries, latency, and the time spent inside each zone. To assess the relative time spent in the center during black box habituation, the time spent in the center zone was divided by the time spent in the periphery. During female preference, the general exploratory activity was calculated by dividing the time spent in both the “Female” and “Dummy” zones by the time spent in the corridor zone.

#### 2.3.2. Sexual behavior

On the fourth day (PND 83), each male rat was placed in a white plastic box (70 × 36 × 55 cm height). Its floor was covered with a layer of clean wood chip bedding. An intact sexually receptive adult female rat was also placed inside the white box (sexual behavior trial). To simulate natural mating conditions, we used intact female rats in estrus. This prevented us from studying intromission rate or ejaculation latency to avoid unwanted pregnancies with respect to the 3R principles. The latency to the first attempted intromission was measured with a maximum trial duration of 10 min. Rats that failed to attempt an intromission were returned to their home cage after 10 min and they were assigned a latency value of 600. An equal percentage of CTR and DER males failed to attempt an intromission.

Video recordings of each trial were analyzed manually, and the total anogenital sniffing time (male to female), total mount attempts, and the time to first attempted intromission during the trial were recorded.

#### 2.3.3. Food anticipation test

To measure the establishment of anticipatory behavior, we designed a two-phase food anticipation task (FA) (based on Patton et al., 2014), where rats had time-restricted access to a limited quantity of standard food chow (two-third of *ad libitum* daily feeding quantity, ~40 g per three rats). For this test, we used an open field arena consisting of a square wooden box with a black painted surface (57 × 57 × 57 cm), with an open top; an empty cylindrical food container (8.5 cm diameter × 0.6 cm height) was placed at a certain corner. To avoid potential behavioral interference, FA experiments were initiated 4 days after the sexual behavior testing of each group. On the first day of the FA test (PND 87), each rat was color marked on its back to facilitate digital tracking, and all food from the home cage was removed. No additional food was added inside the home cage for the rest of the FA days. Before every FA test, all cage mates were weighted and color marked and then simultaneously placed inside the arena. The rats were free to explore the arena for 30 min (context-dependent learning period, **CoP**). At the end of the CoP period, the food container was removed for 2 min; removal of the food container signified the initiation of the cue-dependent learning period (**CuP**). Immediately after the end of the CuP period, the food container filled with 40 g of food was returned to its original position in the arena. The animals were left undisturbed to feed for 30 min inside the arena. At the end of the trial, the rats were returned to their home cage. The arena along with the food container was thoroughly cleaned with 70% ethanol. Leftover food in the arena was retrieved and measured. Water remained available *ad libitum* in the home cages. The food anticipation task took place for 8 consecutive days for each animal group. The first day was designated as day 0 and served as an accommodation trial. Daily weight loss was calculated as the ratio between daily weight and starting weight on day 0 (PND 87) (Dietze et al., 2016). On the final day (day 7, PND 94), the animals were killed immediately after the end of the CuP. The animals were deeply anesthetized using isoflurane vapors, decapitated, and their brains were removed. After removal, the brains were snap-frozen in −35°C isopentane and stored at −80°C. Brains from basal animals of both groups (CTR and DER) were isolated at similar ages.

Every trial was recorded using a digital video camera with auto exposure and autofocus features disabled and zoom manually fixed. EthoVision 7XT (Noldus, Netherlands) software was used to digitally assess recordings of the food anticipation trials. The arena was split into four equal quadrants, and the quadrant containing the food container was designated as **the feeder quadrant**. For multiple animal tracking in food anticipation trial footage, multi-animal tracking setting was applied, and the color marker center point was chosen for animal identification. To smoothen small movement fluctuations, we filtered out movements that were less than 0.5 cm (nesting-minimum distance moved). The software measured the total distance moved and the time spent inside the feeder quadrant. Rearing activity was manually assessed from each recording by three independent observers, and the average was calculated; the feeder quadrant rearing rate was calculated by dividing rearings inside the feeder quadrant by the total rearings measured.

##### 2.3.3.1. Food access priority scoring

To assess the formation and maintenance of a hierarchical food access order, during food restriction, we devised a scoring system (food priority score) that classified each rat according to the order of initial access to the feeder on each day of food anticipation testing. The first rat that reached the feeder was attributed 1 point, the second 2 points, and the third 3 points. The animals were scored accordingly for each trial day. Score values for the 7 days ranged between 7 and 21. By dividing the difference between the maximum and minimum score by 3, we calculated three scoring ranges: a dominant range of 7 to 11.66, an intermediate range from 11.67 to 16.33, and a subordinate range of 16.34 to 21. The animals of each group were distributed to a range according to their total score from all seven recorded trials.

### 2.4. Western blotting

Left hemisphere ventral PFC (anterior–posterior: 3–6.12 mm, rostral to bregma and from dorsal–ventral: 6 mm and below, following olfactory bulb and projection removal) and NAc (anterior–posterior: 1–3 mm) (Paxinos and Watson, 2014) of each animal were homogenized in lysis buffer (100 mM, Tris-HCl pH:7.5, 250 mM NaCl, 48 mM, NaF 2 mM, Na_3_V0_4_, 0.5% Triton X-100, 1:250 protease inhibitor cocktail (Sigma-Aldrich, St. Louis, MO, USA), 2 mM dithiothreitol, 50 mM ethylenediaminetetraacetic Acid), sonicated, and centrifuged at 15,000 rpm for 30 min. The supernatant was collected, and the total protein quantity was determined by 280 nm absorption measured on NanoDrop One (Thermo Fisher). Each Western blot sample was prepared by mixing 65 μg of total protein with NuPAGE lithium dodecyl sulfate (LDS) sample buffer (Thermo Fisher) and 100 mM dithiothreitol (Merck). Samples were heated at 75°C for 20 min and then were loaded and separated on 4–12% gradient NuPAGE Bis-Tris precast polyacrylamide gels (Thermo Fisher) at 0.6A constant current using NuPAGE MOPS running buffer (Thermo Fisher) for 1 h and 30 min. Proteins were then transferred from the gel to a nitrocellulose membrane (0.45 μm nitrocellulose paper Macherey-Nagel, Germany) by applying a constant voltage of 30V for 2 h, using the Surelock-X NuPAGE device and NuPAGE transfer buffer (Thermo Fisher). After transfer, the membranes were incubated in ponceau solution (0.1 mM ponceau (Serva), 0.05% CH_3_C00H) for 1 min to assess transfer efficiency. After ponceau staining, the membranes were washed with ddH_2_O until stained lanes faded. Membrane lanes at 35–40 and 40–50 kDa were blocked in 10% normal goat serum TBS-T (0.05% Triton-X 100) and then incubated with anti-GAPDH mouse monoclonal antibodies, 1:1000 (Millipore) or anti-phosphorylated (ser 133) Cre response element binding protein (pCREB) rabbit polyclonal antibodies, 1:1000 (Millipore), overnight at 4°C. The following day, the membranes were incubated with goat anti-mouse/anti-rabbit polyclonal HRP-conjugated antibodies, 1:100000 (Millipore) for 2 h at room temperature, and luminescence was induced using Immobilon Crescendo (Millipore) substrate. The membranes were exposed to autoradiographic films (Kodak XAR). After exposure, films were developed using Dentus D developer (Agfa, Belgium), fixed using Dentus F (Agfa), washed with dH_2_O, dried, and scanned with a CanoScan8000F scanner. The images were produced by the PhotoStudio 5 (ArcSoft) software using the grayscale method with 600 dpi resolution and saved using the TIF format. Protein band optical density was determined using the ImageJ v.1.53 (NIH) software. pCREB protein levels were assessed relative to GAPDH levels by calculating their ratio (pCREB/GAPDH). The mean of pCREB/GAPDH ratios from CTR samples was used as the reference baseline for each sample of every gel. To compare the groups between conditions, gels containing CTR samples from each condition were used. The mean basal CTR pCREB/GAPDH ratio was used as the baseline and the experimental CTR/basal CTR ratio served as a normalization factor for every experimental measurement.

### 2.5. Statistical analyses

For statistical comparisons, we used the SPSS software (IBM) v.26. For our comparisons, we grouped the animals in groups (CTR vs. DER), litter, condition (basal vs. experimental), and time-point (days) where applicable. Behavioral parameters measured over time were analyzed by generalized estimated equations (GEEs). Each time-point (day) was set as a within-subject factor (repeated measures). In this analysis, we modeled using group as an independent factor, group (litter) as a nested factor, day as a repeated measure, and the interaction of group × day, followed by *post hoc* Bonferroni sequential test when appropriate. In every GEE comparison, the food priority score and eight loss were factored in as covariates. We considered that food access priority influenced the behavioral output of the next day (Bruce, 1941). Consequently, the daily food priority score was factored in as a covariate in the analysis of the behavior of the next day. If group × day interactions were found, we performed *post hoc* GLM for each time-point separately with group and group (litter) modeled together with food priority score and weight loss factored in as covariates. To identify hierarchical alterations in each group during the food anticipation trials, we applied the chi-square test. The CTR group distribution in the three food scoring ranges was set as the expected distribution, and the distribution of the DER group was observed. To identify the potential effects of the DER experience on pCREB protein levels during the experimental manipulations, we used a generalized linear model (GLM), where we modeled using an independent factors group and condition as well as their interaction group × condition and as a nested factor group (litter). When group × condition interaction was found, *post hoc* Bonferroni sequential comparisons were made between groups for each condition (basal CTR vs. basal DER, and experimental CTR vs. experimental DER) and between conditions for each group (basal CTR vs. experimental CTR, and basal DER vs. experimental DER). For all tests, differences were considered significant when a p-value = < 0.05.

## 3. Results

### 3.1. Female preference and sexual behavior: the DER experience does not impact the sexually motivated socialization or the initiation of coitus of male rats

The female preference trial investigated the DER effect on the sexually driven socialization of naïve male rats when faced with an unfamiliar sexually receptive female rat. In order to correctly interpret any results obtained for sexually driven socialization, it was deemed necessary to determine any stress-related confounding effects of novelty exposure to the test arenas: GLM analysis of the black box habituation behaviors showed that there was no difference in total distance moved (DER:1338.96 cm vs. CTR:1348.47 cm, W = 0.004, *p* = 0.948), time spent in center/time spent in the periphery (DER:0.0041 vs. CTR:0.0063, W = 0.948, *p* = 0.330), or center zone number of entries (DER: 0.733 vs. CTR:1.44 sec, W = 0.32, *p* = 0.074), indicating that the arena novelty affected similarly both groups. Additionally, GLM analysis showed no difference between CTR and DER animals in total distance moved (DER: 2589.36 cm vs. CTR: 2169.07 cm, W = 0.071, *p* = 0.79), total entries committed in female and dummy compartments (DER: 12.91 vs. CTR: 11.85, W = 0.55, *p* = 0.46), or female+dummy/corridor time spent ratio (DER: 18.47 vs. CTR: 5.56, W = 2.006, *p* = 0.157). Furthermore, there was no difference in female compartment entry latency (DER: 30.53 sec vs. CTR: 36.65 sec, W = 0.34, *p* = 0.56) or dummy compartment entry latency (DER: 187.27 vs. CTR: 261.33, W = 0.51, *p* = 0.48). These results suggest that the DER experience did not affect the typical exploratory activity in the presence of an unknown sexually receptive female or an inanimate rat-like object. In female preference trials both CTR and DER animals spend more time around (DER: 65.9% vs. CTR: 64.5%, W = 0.071, *p* = 0.79) and actively explored through sniffing (DER: 75.1% vs. CTR: 80.7%, W = 2.378, *p* = 0.123) the cage containing the receptive female compared to the cage containing the rat-like dummy.

The sexual preference trial measures the natural manifestation of sexual behaviors by naïve male rats. There is no difference between CTR and DER animals regarding the time required to initiate copulation (CTR: 370.4 sec vs. DER: 398,44 sec W = 0.25, *p* = 0.62). Additionally, there appears to be no difference in the precopulatory behaviors as both anogenital sniffing (% of the total time spent with the female, CTR: 12.6% vs. DER: 11.16% W = 0.6, *p* = 0.437) and mount attempts (CTR: 2.13 vs. DER: 2.22 W = 0.017, *p* = 0.895) before the first intromission were similar in the two groups. These results suggest that the DER experience does not affect the sexual drive or copulation initiation of male rats.

### 3.2. Food anticipation: the DER experience alters the preference for the area where the food reward is expected to be delivered

The food anticipation (FA) trials explore the effects of the DER experience on the anticipatory behavior of the animals when a reward in the form of food is expected to be delivered. These trials are designed to record the learning progression and efficiency of two distinct learning modules: contextual and cue learning. Thus, each trial initiates with a 30-min context-dependent learning period followed by a 2-min cue-dependent learning period where the removal of the empty food container acts as a reward-predicting cue.

#### 3.2.1. Context-dependent learning

**Figure 2:**
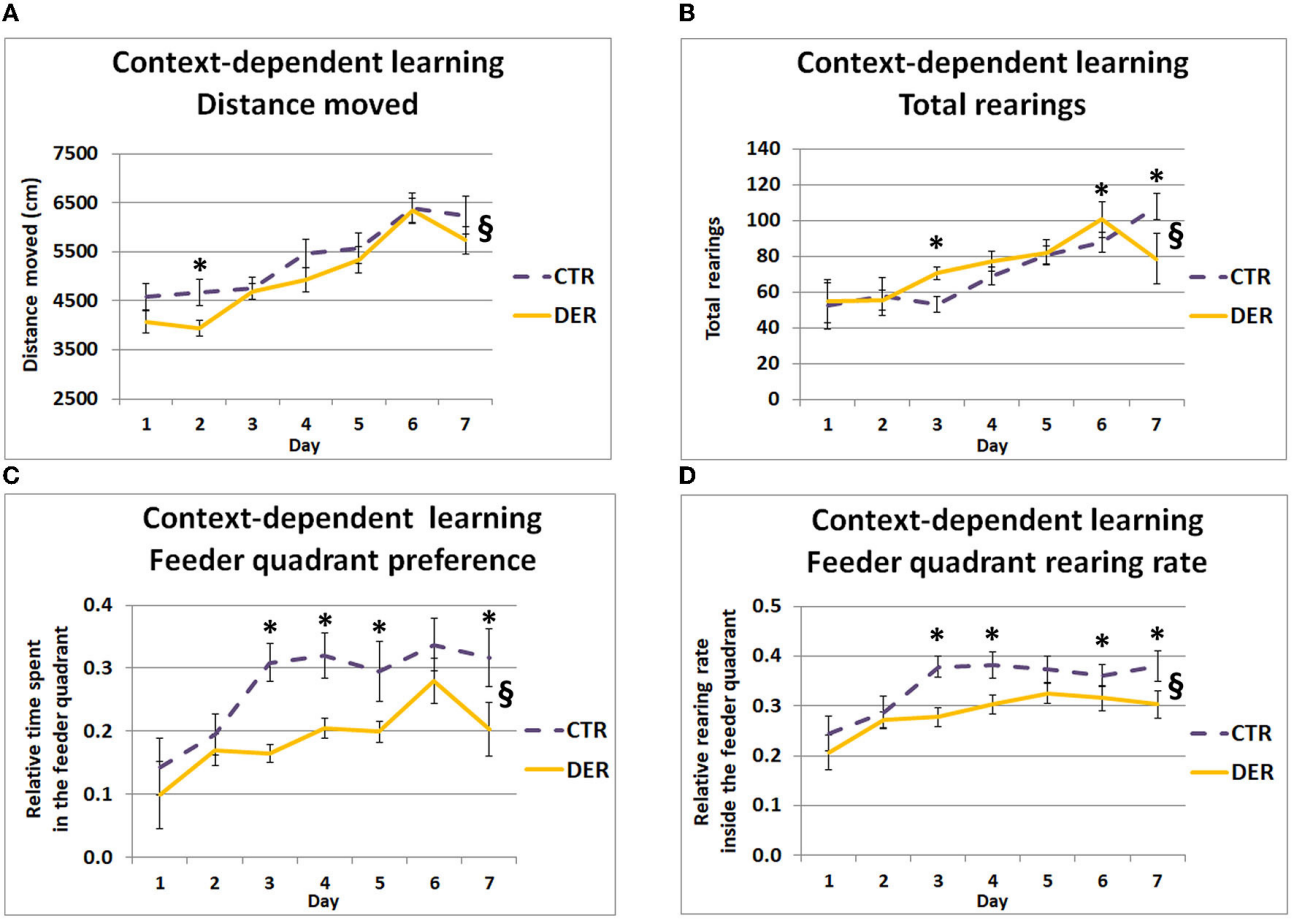
Behavior during the context-dependent learning period of food anticipation (FA) training. **(A)** Total distance moved (cm) in the open field arena during the 30-min context-dependent learning period for each day (one trial/day). On day 2 of FA training, DER animals showed reduced mobility. Lines represent means ± SEM. (CTR: *n* = 18, DER: *n* = 18). §: GEE group × day interaction, *p* < 0.05; ^*^: *post hoc* group effect, *p* < 0.05. **(B)** Total rearings performed during the 30-min context-dependent learning period for each day (one trial/day). On days 3 and 6, DER animals displayed increased rearing activity, while on day 7 this was lower, compared to CTR. Lines represent means ± SEM. (CTR: *n* = 18, DER: *n* = 18). §: GEE group × day interaction, *p* < 0.05; ^*^: *post hoc* group effect, *p* < 0.05. **(C)** Relative time spent in the rewarded quadrant of the open field (quadrant containing the empty food container in which food will be provided after the end of the anticipation training), during the 30-min context-depended learning period for each day (one trial/day). Relative time was calculated by dividing the time spent inside the rewarded quadrant by the total time of the context-dependent learning period. DER animals preferred less the rewarded quadrant on days 3, 4, 5, and 7 of FA training compared to CTR. Lines represent means ± SEM (CTR: *n* = 18, DER: *n* = 18). §: GEE group × day interaction, *p* < 0.05; ^*^: *post hoc* group effect, *p* < 0.05. **(D)** Rearing rate inside the rewarded quadrant of the open field (quadrant containing the empty food container in which food will be provided after the end of the anticipation training), during the 30-min context-depended learning period for each day (one trial/day). Rearing rate was calculated by dividing rearings inside the feeder quadrant by the total rearings measured. DER animals displayed a reduced rearing rate inside the rewarded quadrant on days 3, 4, 6, and 7 of FA training compared to CTR. Lines represent means ± SEM (CTR: *n* = 18, DER: *n* = 18). §: GEE group × day interaction, *p* < 0.05; ^*^: *post hoc* group effect, *p* < 0.05.

Studying mobility during the context-dependent learning period (**CoP**) can reveal critical components of anticipatory behaviors such as hypermobility and increased exploration of the area where food is expected to be delivered. The changes in the distance moved and rearing activity across the days of FA training can be correlated with anticipatory responses. GEE covariate analysis showed a significant daily weight loss effect on total distance moved (W = 6.76, *p* = 0.009). Additionally, GEE analysis of the total distance moved and rearing activity during the CoP revealed significant group × day interactions (W = 21.75, *p* = 0.001 and W = 46.85, *p* < 0.001), indicating horizontal and vertical locomotion differences between the groups on certain training days. *Post hoc* analysis of the total distance moved showed that the DER group displayed lower locomotion on day 2 (W = 5.29, *p* = 0.021) compared to the CTR group (Figure 2A). *Post hoc* analysis of the total rearings indicated that the DER group displayed higher rearing activity on days 3 (W = 14.95, *p* < 0.001) and 6 (W = 6.1, *p* = 0.013), while lower rearing activity on day 8 (W = 7.82, *p* = 0.005) compared to CTR (Figure 2B). In the following days, general locomotion reached similar levels, increasing steadily on each training day.

Increased time spent and rearing rate inside the quadrant where the empty food container is located can act as an indicator of context-reward association. GEE covariate analysis showed a significant food priority score effect both on relative time spent (W = 6.96, *p* = 0.008) and rearing rate (W = 5.66, *p* = 0.02) inside the feeder quadrant. Moreover, GEE analysis showed a group × day interaction on the relative time spent (W = 25.93, *p* < 0.001) and rearing rate (W = 13.19, *p* = 0.04) in the quadrant where food was later delivered, indicating a difference between the groups in the development of preference and targeted exploration for the quadrant where food delivery was expected: DER rats' preference for and rearing rate in the rewarded quadrant appeared lower than normal. *Post hoc* analysis of the relative time spent in the feeder quadrant, for each day, indicated that DER animals spent less time in the quadrant where food was delivered on days 3 (W = 17.86, *p* < 0.001), 4 (W = 9.40, *p* = 0.002), 5 (W = 4.67, *p* = 0.031), and 7 (W = 11.349, *p* < 0.001) (Figure 2C), suggesting an impaired context-reward association by the DER rats. Similarly, *post hoc* analysis of the rearing rate in the feeder quadrant showed lower rate by the DER animals on days 3 (W = 15.16, *p* < 0.001), 4 (W = 6.13, *p* = 0.013), 6 (W = 4.0 5, *p* = 0.044), and 7 (W = 7.7, *p* = 0.006) (Figure 2D). Interestingly, while DER animals did not exhibit major alterations on general anticipatory mobility and exploratory activity, they displayed lower preference and exploration rate of the quadrant containing the empty food container.

#### 3.2.2. Cue-dependent learning

**Figure 3:**
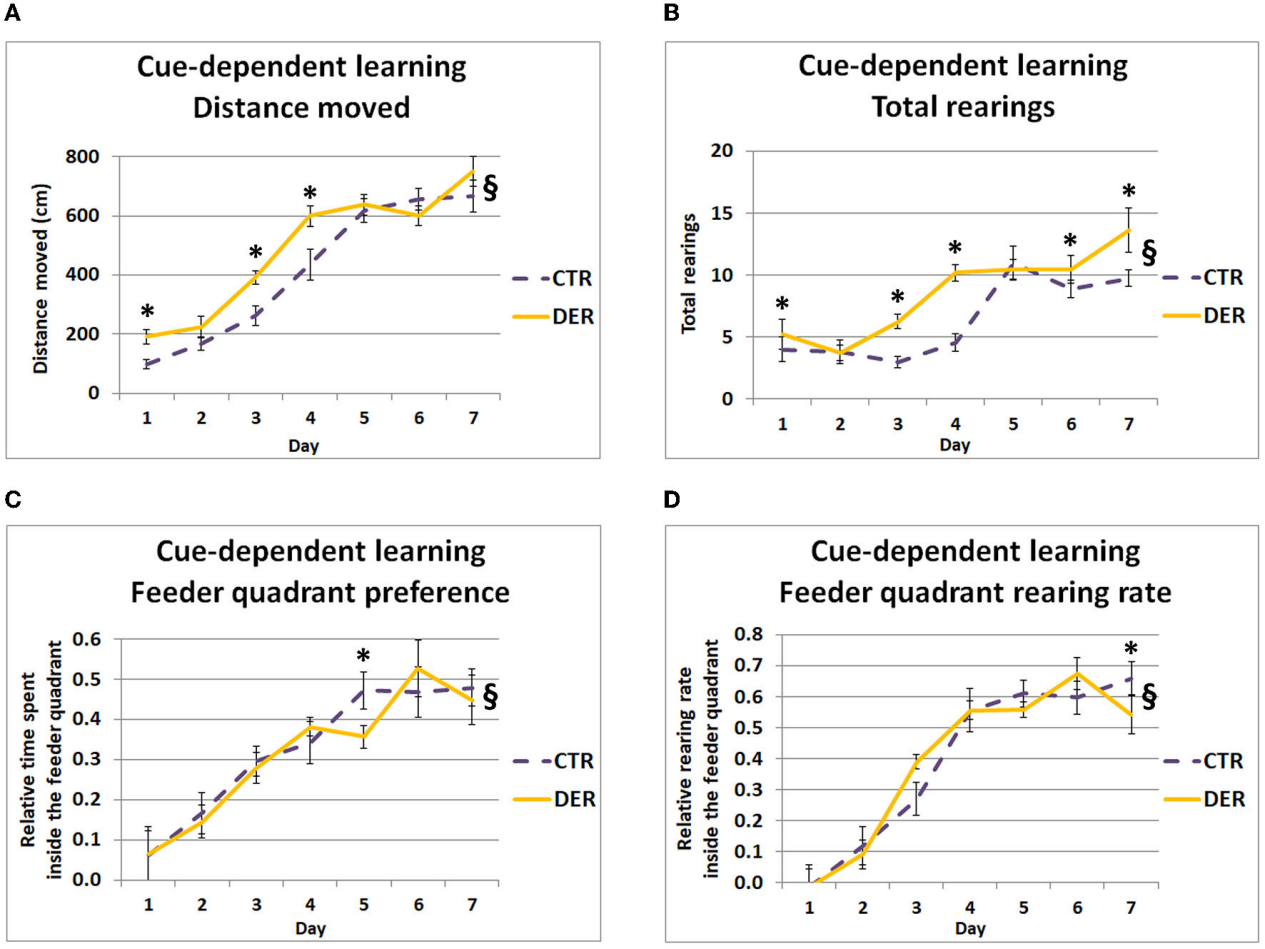
Behavior during the cue-dependent learning period of food anticipation (FA) training. **(A)** Total distance moved (cm) during the 2-min cue-dependent learning period for each day (one trial/day). DER animals showed increased mobility on days 1, 3, and 4. Lines represent means ± SEM. (CTR: *n* = 18, DER: *n* = 18). §: GEE group × day interaction, *p* < 0.05; ^*^: *post hoc* group effect, *p* < 0.05. **(B)** Total rearings performed during the 2-min cue-dependent learning period for each day (one trial/day). DER animals displayed increased rearing activity on days 1, 3, 4, 6, and 7 compared to CTR. Lines represent means ± SEM. (CTR: *n* = 18, DER: *n* = 18). §: GEE group × day interaction, *p* < 0.05; ^*^: *post hoc* group effect, *p* < 0.05. **(C)** Relative time spent on the rewarded quadrant of the open field (quadrant where food will be provided after the end of the anticipation training), during the 2-min cue-depended learning period for each day (one trial/day). Relative time was calculated by dividing the time spent inside the rewarded quadrant by the total time of the cue-dependent learning period. DER rats preferred less the rewarded quadrant on day 5. Lines represent means ± SEM. (CTR: *n* = 18, DER: *n* = 18). §: GEE group × day interaction, *p* < 0.05; ^*^: *post hoc* group effect, *p* < 0.05. **(D)** Rearing rate inside the rewarded quadrant of the open field in which food delivery is expected, during the 2-min context-depended learning period for each day (one trial/day). Rearing rate was calculated by dividing rearing inside the feeder quadrant by the total rearings measured. DER animals displayed a reduced rearing rate inside the rewarded quadrant on day 8 compared to CTR. Lines represent means ± SEM (CTR: *n* = 18, DER: *n* = 18). §: GEE group × day interaction, *p* < 0.05; ^*^: *post hoc* group effect, *p* < 0.05.

Studying the behavioral responses following the removal of the food container (**CuP)** allowed us to investigate the DER effects on cue-reward conditioning (Pavlovian dependent–independent cue association). GEE covariate analysis indicated a significant daily weight loss effect both on distance moved (W = 4.16, *p* = 0.041) and total rearings during CuP (W = 7.42, *p* = 0.006), suggesting that weight loss influenced general anticipatory mobility and exploratory activity. In addition, GEE analysis revealed a group × day interaction both on the distance moved (W = 34.59, *p* < 0.001) and total rearings (W = 36.82, *p* < 0.001), suggesting differential mobility and exploratory activity between the groups following the cue onset. *Post hoc* analysis showed that DER animals displayed increased distance moved on days 1 (W = 10.66, *p* = 0.001), 3 (W = 10.33, *p* = 0.001), and 4 (W = 7.08, *p* = 0.008) (Figure 3A). Furthermore, *post hoc* analysis showed that the DER group displayed increased rearing activity on days 1 (W = 7.32, *p* = 0.007), 3 (W = 30.827, *p* < 0.001), 4 (W = 37.62, *p* < 0.001), 6 (W = 4.66, *p* = 0.031), and 7 (W = 6.66, *p* = 0.01) (Figure 3B). DER rats appeared to respond with increased horizontal mobility after the removal of the feeder (cue) on the first days of FA, while after day 5, horizontal mobility levels were similar in both groups. Interestingly, DER animals displayed increased rearing activity on almost every FA test day suggesting a reinforced general exploratory response to the reward-predicting cue.

Regarding the relative time spent and the rearing rate inside the rewarded quadrant, GEE covariate analysis showed a significant food priority score effect on both parameters (W = 9.68, *p* = 0.002 and W = 10.04, *p* = 0.001). Furthermore, GEE analysis of the relative time spent and rearing rate inside the feeder quadrant revealed a group × day interaction (W = 17.13, *p* = 0.009 and W = 22.29, *p* < 0.001, respectively), suggesting a difference in the association rate of the quadrant where food was provided after the end of the anticipation period with the imminent food delivery. *Post hoc* analysis revealed that the DER rats spent less time within the rewarded quadrant on day 5 (W = 5.46, *p* = 0.019) (Figure 3C) and showed a lower rearing rate inside this quadrant on day 8 (W = 6.42, *p* = 0.011) compared to the CTR (Figure 3D). While in both groups preference for the rewarded quadrant gradually increased, on day 5, DER animals exhibited a delay in their preference increase. Nevertheless, from day 6, DER animals achieved a similar preference with the CTR. Notably, on day 8, the DER rearing rate inside the rewarded quadrant was lower than that of the CTR ones.

### 3.3. The DER experience induces a disturbance in the food access priority during group FA trials

**Figure 4:**
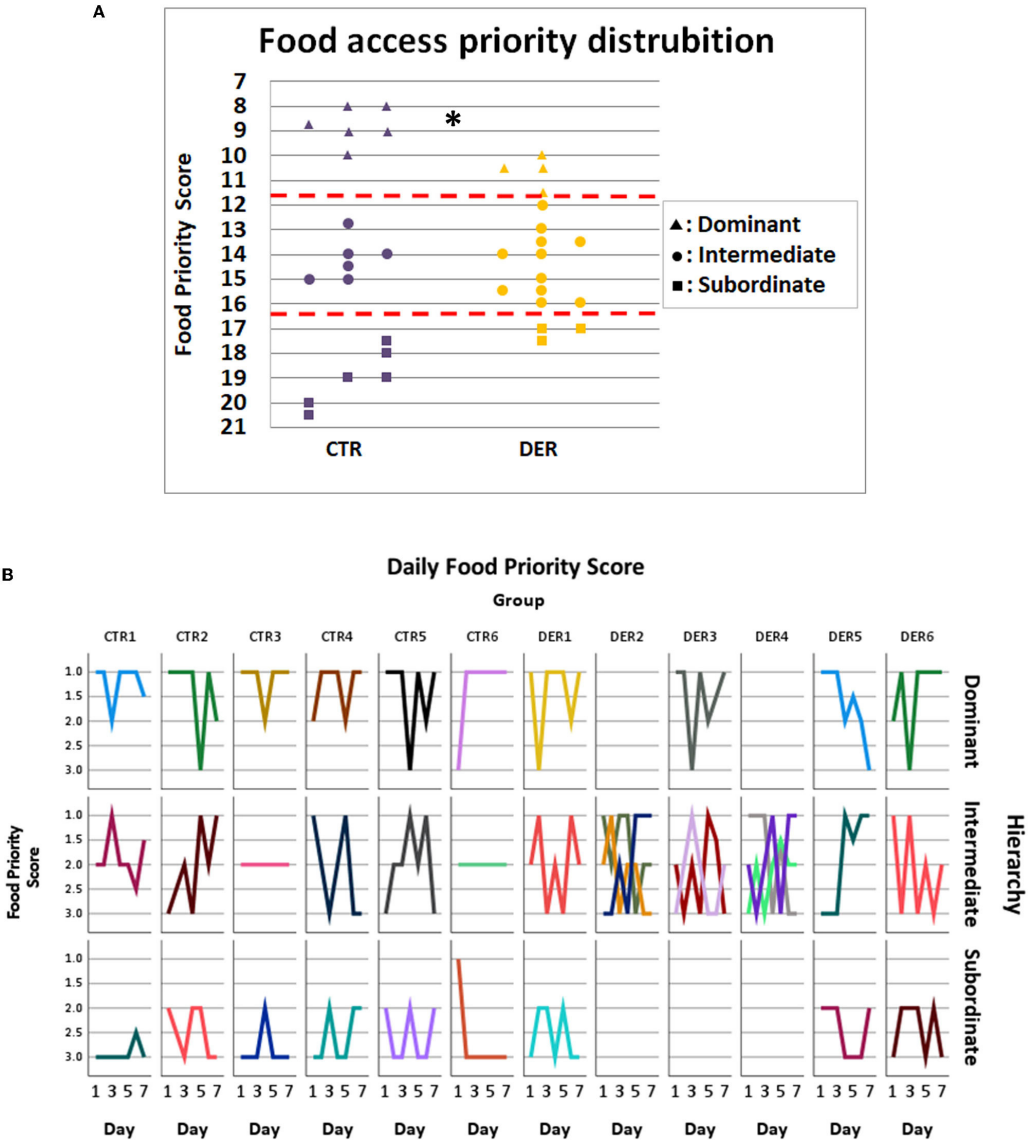
Food access priority analysis. **(A)** Food priority score of each animal from each group. Each animal was categorized according to its total food priority score (FPS) from a total of seven trials: dominant: FPS < 11.66, intermediate: 11.66 < FPS < 16.33, and subordinate: FPS > 16.33. (CTR: *n* = 18, DER: *n* = 18). ^*^: Chi-square *p* < 0.05. **(B)** Each line depicts the daily food priority scores of an individual animal. On each column, all cage mates of a group are presented, while on each row animals of the same overall hierarchical category (dominant, intermediate, and subordinate) are presented. Empty subplots indicate that there was no appropriate animal among the cage mates for the given hierarchical position.

The FA was conducted as a group task where triads of cage mates learned to anticipate and receive food rewards after a set period. Stable food access priority could indicate an established social hierarchy. To assess food access priority, the access order to the food reward for every animal on each triad was scored, and the total score (food priority score) determined its position as dominant, intermediate, or subordinate. DER score distribution was significantly different (Chi2 = 6.273, *p* = 0.043) compared to that of the CTR, with more animals categorized as intermediate, an indication of an unstable reward access priority (Figure 4).

### 3.4. DER experience increases pCREB (ser 133) protein levels in vPFC and NAc following 8 days of FA

**Figure 5:**
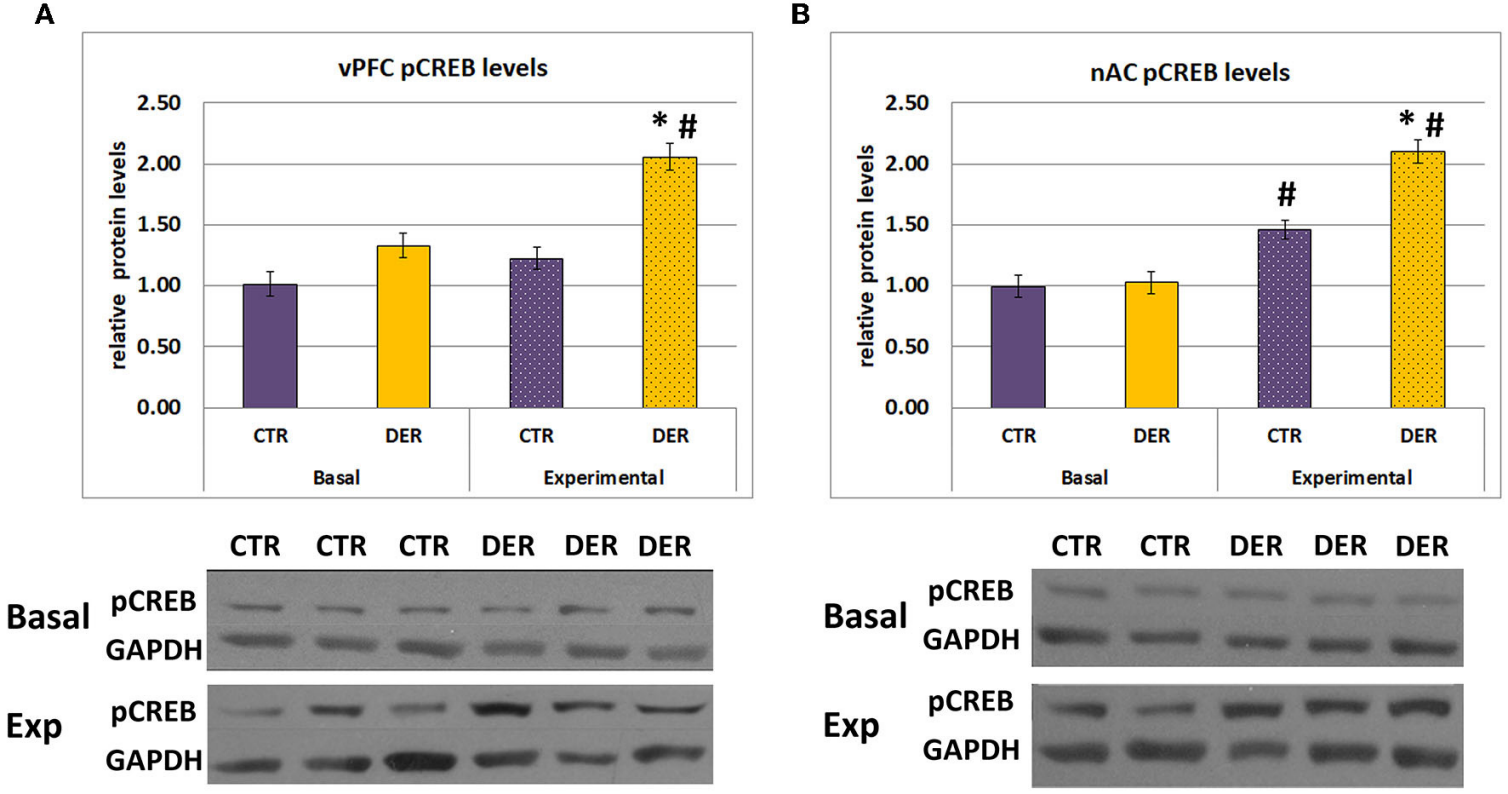
Relative protein levels determined by Western blotting. Protein levels were normalized using the basal CTR group as the baseline 100%. **(A)** PFC relative pCREB protein levels, normalized to the CTR basal group. DER animals showed increased pCREB levels following exposure to the FA training for 8 days. Bar graphs represent means ± SEM. (basal CTR: *n* = 17, basal DER: *n* = 18, experimental CTR: *n* = 23, experimental DER: *n* = 15). ^*^: *post hoc* group effect (experimental CTR vs. experimental DER), *p* < 0.05, #: *post hoc* condition effect (basal DER vs. experimental DER), *p* < 0.05. **(B)** NAc relative pCREB protein levels, normalized to the CTR basal group. Both DER and CTR animals showed increased pCREB levels under experimental conditions. Furthermore, exposure to the FA training for 8 days caused a stronger pCREB activation in DER animals. Bar graphs represent means ± SEM. (basal CTR: *n* = 17, basal DER: *n* = 18, experimental CTR: *n* = 24, experimental DER: *n* = 15). ^*^: *post hoc* group effect (experimental CTR vs. experimental DER), *p* < 0.05; #: *post hoc* condition effect (basal CTR vs. experimental CTR and basal DER vs. experimental DER), *p* < 0.05. Sample immunoblotting images are displayed for each group. pCREB bands appeared at 47 kDa and GAPDH bands appeared at 37 kDa.

FA results indicated that the DER experience induces reward association deficiencies. To identify potential DER effects on the reward circuit activation during the FA trials, we measured CREB phosphorylation (ser 133) status, an indicator of neuronal activation, in the ventral prefrontal cortex (vPFC), and in the nucleus accumbens (NAc). GLM analysis of vPFC pCREB levels showed a group × condition statistically significant interaction in both vPFC (W = 6.83, *p* = 0.009) and NAc (W = 11.518, *p* < 0.001) (Figure 5). Furthermore, *post hoc* analysis of vPFC pCREB levels indicated that only the DER group showed increased pCREB levels immediately after the final FA trial compared to the basal conditions (basal CTR vs. experimental CTR: *p* = 0.06, basal DER vs. experimental DER: *p* < 0.001) (Figure 5A). Moreover, pCREB levels were elevated only in the experimental DER group compared to the respective controls (basal CTR vs. basal DER: *p* = 0.07, experimental CTR vs. experimental DER: *p* < 0.001, Figure 5A).

Further *post hoc* analysis of NAc pCREB levels showed that pCREB levels were elevated in both groups immediately after the final FA trial (basal CTR vs. experimental CTR: *p* < 0.001, basal DER vs. experimental DER: *p* < 0.001) (Figure 5B). Interestingly, while basal NAc pCREB levels were similar between the two groups, experimental DER pCREB levels were significantly higher compared to experimental CTR (basal CTR vs. basal DER basal: *p* = 0.82, experimental CTR vs. experimental DER: *p* < 0.001) (Figure 5B). These results suggest that the DER experience induced pCREB overactivation in both vPFC and NAc during reward anticipation.

## 4. Discussion

In the present study, we studied the effects of the DER experience on two innately rewarding conditions for adult male rats: contact with a sexually receptive female and food anticipation during food restriction. Initially, we showed that there were no significant differences in behaviors indicating anxiety/stress response between DER and CTR rats when singly exposed to the novel environments of an open field or of a three-compartment setup employed for the study of receptive female socialization. We also found no differences regarding the sexually driven socialization or initiation of mating behaviors in the presence of a sexually receptive female between DER and CTR animals. Interestingly, we did find alterations in anticipatory behaviors, food access order stability, and brain activity of DER rats following group FA training. Food deprivation and the resulting weight loss affected horizontal locomotion during the context-dependent learning phase (CoP) as well as both horizontal and vertical ambulatory activity during the cue-dependent learning phase (CuP). On the other hand, food access priority affected both the relative time spent and the rearing rate within the feeder quadrant during both phases (CoP and CuP). In addition to these effects, the DER experience affected on specific days of FA training the four parameters defined; the major finding being a context-reward association deficit of the DER animals. This erratic reward association of the DER animals was accompanied by social instability of food access order following food presentation. Interestingly, Western blot analysis immediately after the final day of FA training revealed increased expression of pCREB (ser 133) in both vPFC and NAc of DER rats compared to CTR. Collectively, these results highlight a negative effect of the DER experience on the acquisition of reward preference, social access stability, and cellular signaling during reward anticipation.

Our results showed that the DER experience did not affect sexually driven socialization or the initiation of copulatory behaviors of naive adult male rats. Specifically, DER rats explored and spent a similar amount of time compared to CTR near the area where sexually receptive females were located. Furthermore, anogenital sniffing, mount frequency, and latency to the first intromission in the sexual preference trials were similar between DER and CTR animals. To probe for potential exploratory deficits or neophobic responses that could interfere with our results, open field and three-compartment exploration analyses were conducted and showed that DER animals responded to novelty similarly to CTR. What we cannot rule out with our experimental setup is any mild impairment in DER male responses when faced with females in different estrous cycle phases. Despite the limited range of our measurements, our findings were comparable to reports from other studies measuring similar behavioral responses. Although our hypothesis that increased aggression and play-fight displayed by the DER rats (Diamantopoulou et al., 2012) could also affect sexually driven behaviors, our findings dissociate DER social aggression from the sexual drive (Veening et al., 2005). Available information concerning the effects of early life adversity on the sexual drive of rats remain inconclusive and often controversial. One study showed that 6h maternal separation on PND 2–10 induced increased mating latency (Rhees et al., 2001), while another study in which rats were separated from their mothers for 3 h from PND 2 to 14 showed lower mounting and intromission latency (Greisen et al., 2005). Another study of early life adversity using limited bedding from PND 2 to 10 found an increased male preference for sexually receptive females but no impact on mounting or intromission latency (Davis et al., 2020). As our findings are not in-line with these studies, it appears that the DER phenotype does not disrupt motivational or consummatory aspects of naïve male sexual behavior, highlighting it as a unique early life stress paradigm with distinct phenotypical attributes.

The food anticipation (FA) training is essentially an open field arena (Gould et al., 2009) containing an unfamiliar object (feeder) placed at a fixed corner. Findings from the context-dependent learning period (CoP) of the grouped FA training showed that the DER animals displayed mild locomotion and exploratory behavior alterations, and, generally, increased avoidance of the quadrant containing the empty feeder compared to CTR. It is known that unfamiliar novel objects induce avoidance responses in rats (Corey, 1978), and that early life stress could augment the expression of such responses (Meaney et al., 1994). Additionally, reduced mobility levels in open field trials have been reported following social stress in normal (Rygula et al., 2005) and DER rats (Diamantopoulou et al., 2018). Considering that the DER experience induced passive behavioral responses under stressful conditions (Diamantopoulou et al., 2012), we can infer that the behavioral responses of the DER animals of decreased horizontal mobility on day 2 and decreased vertical mobility on day 7 of FA training were caused by increased stress. The potential stressors in our FA trials include, apart from the novel environment, food deprivation, and possibly social factors. Food restriction, an internal component of FA training, leads to weight loss and enhanced motivational drive for food rewards (Tapp et al., 2020) but can also induce stress responses (Heiderstadt et al., 2000; Tomiyama et al., 2010). Counterintuitively, the presence of conspecifics could also confer to the stressful nature of FA training: Although social interactions normally reduce stress (Lemos et al., 2021), they can also induce stress transmission in a process called stress contagion (Carnevali et al., 2017, 2020). In support of the influence of a socially related stressor, our findings from the black box habituation and previous experiments in our lab employing open field assays (Diamantopoulou et al., 2012) indicated no ambulatory activity alterations of individually tested, not under previous stress, DER animals. All these stressors (novel object, food deprivation, and social stressor), combined, could act synergistically on the animals leading to the reduced mobility and feeder quadrant avoidance exhibited by the DER animals. These observations provide novel information concerning the adverse effects of the DER experience during novelty exposure in groups.

Horizontal locomotion levels from the third day onward did not differ between DER and CTR animals and in both groups, they increased progressively, as did the number of rearings, suggesting that the FA training could effectively induce anticipatory hyperactivity in both groups. Indeed, we showed that horizontal mobility levels were influenced by weight loss, and previous reports have associated food deprivation with elevated locomotion (Murphy and Nagy, 1979; Timberlake and White, 1990). Elevated locomotion could also be explained by circadian food entrainment although such effects are typically observable after a week of food restriction (Boulos et al., 1980; Webb et al., 2009; Patton et al., 2014). Notably, at the same timeframe, both DER and CTR feeder preference levels increased but contrary to locomotion, DER animals generally spent less time and displayed lower rearing rates inside the feeder quadrant compared to CTR. An interesting observation is that the DER rearing rate inside the feeder quadrant was lower than CTR even on days when DER animals displayed increased total rearings. This indicates that although FA training could effectively induce hyperactivity and reward association in agreement with other reports (Carr, 2002; Silver et al., 2011), the DER experience interfered with reward-context association. Contextual memory impairments have also been described in other models of early life adversity (Kosten et al., 2012; Loi et al., 2017). Loi linked increased hippocampal glucocorticoid receptors with contextual memory deficiency that was reversed after glucocorticoid receptor inhibition on PND 26–28. Notably, DER rats also display increased hippocampal glucocorticoid receptor density (Diamantopoulou et al., 2012). Interestingly, contextual memory formation is dependent on hippocampal interaction with NAc and mPFC (Yang and Liang, 2014) highlighting these areas as potential mediators of the abnormal reward response observed in DER animals. Alternatively, it has been reported that stress can trigger depression-like mood disorders potentially affecting reward learning (Cléry-Melin et al., 2019) and disrupting associative memory as observed in humans (Goldfarb et al., 2019) and animals (De Quervain et al., 1998; Sunanda et al., 2000; Moreira et al., 2016). The stress of novelty discussed previously combined with food restriction stress could induce the observed DER-driven reward associative memory deficiency. Additionally, the statistical analysis highlighted food priority access as an important factor affecting the development of feeder quadrant preference. Thus, the increased social instability (discussed below) could confer to the etiology of the reward association deficits observed in DER animals during the CoP, as a social conflict for a limited resource is a well-known stressor (Hodge et al., 2009).

During the cue-dependent learning period (CuP), DER animals displayed increased locomotion compared to CTR on days 1, 3, and 4, as well as increased rearing activity on most days following feeder removal (cue). Additionally, we found that both the distance were moved, and total rearings were also influenced by daily weight loss. Conversely, DER preference for the quadrant where the food was expected to be delivered was lower on day 5, and the rearing rate inside the same quadrant was lower on day 8. Analogous to the CoP, both behaviors were influenced by food priority scores. Again, horizontal and vertical locomotion levels increased progressively for both groups. Both groups responded to the food-predicting cue developing behavioral responses that closely resemble Pavlovian conditioning (Fitzpatrick and Morrow, 2016), namely increased locomotion and reward place preference following cue presentation. The increased general mobility of the DER rats observed on the first day could reflect stress-relieving effects following the removal of the feeder. On the other hand, the higher locomotion of DER animals on later days as well as the increased rearing activity could result from a reward over-sensitization due to food restriction (Abrahamsen and Carr, 1996; Tapp et al., 2020). Notably, locomotor sensitization has been proposed as a predictor of drug addiction (Robinson and Berridge, 2008), and locomotion and place preference sensitization are both considered indicators of drug abuse induced by early life stress (Levis et al., 2022), as early life adversity has been associated with increased risk for substance abuse in both humans (Enoch, 2011; Varese et al., 2012) and rodents (Levis et al., 2022). Despite their general vertical and horizontal locomotor over-sensitization, DER rats displayed delayed preference acquisition and lower rearing rate targeted to the rewarded quadrant on day 8, compared to CTRs, indicating a reward place association deficiency. It should be noted that DER quadrant preference reached CTR levels after day 6, and the rearing rate fell below CTR levels on day 8, despite their higher total rearing count. Collectively, these results indicate that although DER animals learned to expect the imminent arrival of the cue-predicted reward, they had difficulty forming a spatial-reward association. Similar effects were observed in a two-hit study where maternal separation and stress induction caused short-term spatial memory disruption (Hill et al., 2014). This further supports the hypothesis that the spatial deficiency induced by the DER experience during both CoP and CuP of FA training could be stress related. An alternative explanation could be that the elevated stress of the DER animals led to the development of an alternative reward-driven strategy favoring increased exploratory activity instead of a reward area-focused response. Such effects have been observed in mice where acute stress induced a shift from a spatial-focused strategy to a stimulus response one (Schwabe et al., 2010).

FA training is a group task where three cage mates learn together to anticipate food rewards. Social stability maintained through hierarchy can reduce severe conflicts and aggression (Curley, 2016). Conversely, antagonistic or aggressive behaviors during FA training, such as those observed in DER rats (Diamantopoulou et al., 2012), could interfere with reward learning. Indeed, many reports have correlated social stress with reward learning disruption (van der Harst et al., 2005; Kamal et al., 2010; Kúkel'ov et al., 2018). The inability to form a stable hierarchical structure could lead to increased competition during reward receipt disrupting normal reward memory association. Food access competition has recently been found to be a fitting marker of the dominance status in rats (Costa et al., 2021). In the present study, we analyzed food access order and showed that while each CTR animal maintained a rather stable food access hierarchy, DER rats in groups of three frequently interchanged food access orders on successive FA training days, suggesting that DER animals display elevated competitive behavior. Other reports on early life adversity are controversial: One study showed that mice that had undergone a maternal separation, displayed subordinate behaviors during a water receipt task (Benner et al., 2014), while another study showed that maternally separated rats displayed augmented social winning in tube tests and elevated food-receipt dominance (Tada et al., 2016). Importantly, many studies have shown that the medial prefrontal cortex (Benner et al., 2014; Zhou et al., 2017; Padilla-Coreano et al., 2022) and NAc (Shan et al., 2022) are implicated in determining the hierarchical status of animals during social competition tasks. Collectively, our findings highlight a previously undescribed effect of the DER experience on social stability while they also suggest a neural link between social dynamics and reward prediction-learning which could underlie the behavioral abnormalities observed in DER animals.

To assess reward circuit activation during reward anticipation, we determined pCREB levels in NAc and vPFC, two brain regions highly correlated with reward learning and motivated responses (Salamone and Correa, 2002; Hiser and Koenigs, 2018). CREB is a transcription factor that becomes active when phosphorylated by various kinases such as PKA, Ca2+/calmodulin-dependent protein kinases, mitogen-activated protein kinases (MAPKs), and other kinases on the serine 133 residue (Alberini, 2009). Being a central downstream mediator of many signaling pathways, phosphorylated CREB (ser 133) is an ideal marker of neuronal activation (Wang et al., 2018). We found that under basal conditions, DER and CTR adult animals displayed similar pCREB levels in both NAc and vPFC. Notably, in vPFC, pCREB levels were increased only in the DER group following 8 days of FA training. This finding is unexpected given the contextual memory impairment and unfocused cue-induced exploration of DER animals, as in literature, pCREB increases in the PFC have been considered an indication of enhanced memory performance in rodents (Hotte et al., 2006; Cho and Han, 2016). Nevertheless, there are studies suggesting that PFC activation is not always beneficial. A study in humans implicated elevated brain activation during social competition with a reduction in working memory performance (DiMenichi and Tricomi, 2017). Moreover, hyperactivation of ventromedial PFC was detected in humans with obsessive-compulsive disorders (Stern et al., 2013; Apergis-Schoute et al., 2018) or in children with ADHD during a multi-source interference task (MSIT) (Zamorano et al., 2017). In any case, PFC function has been associated with the facilitation of reward-seeking behaviors (Caballero et al., 2019) and especially context-dependent responses (Moorman and Aston-Jones, 2015), and we can postulate that DER animals show a protracted PFC activation compared to CTRs as they are still forming on the eighth day of FA neuronal associations between environmental cues and reward receipt.

The FA training elicited increased pCREB levels in the NAc of both DER and CTR rats. Interestingly, this increase was larger in DER animals. CREB activation in NAc can be triggered by many stimuli, rewarding or aversive. Consequently, NAc functional activation has been linked to a variety of emotional responses that are often contradictory. Indeed, some studies reported that chronic pCREB activity in NAc can lead to anhedonia, while the inhibition of CREB function in NAc promotes reward (Barrot et al., 2002; Carlezon et al., 2005). The functional dichotomies observed in NAc activation lie in its anatomical specificity. For example, NAc shell activation induced elevated locomotion during reward presentation, while NAc core activation produced the opposite effect (Tzschentke and Schmidt, 2000). Typically, pCREB activation in NAc has been correlated with increased reward learning (Hart et al., 2014), punishment aversion (Qi et al., 2016), and long-term memory formation (Schotanus and Chergui, 2008; Wolf and Ferrario, 2010; for an extensive review see Bayassi-Jakowicka et al., 2021). On the other hand, overactivation of the NAc has been linked to pathophysiologic conditions such as schizophrenia (Wang et al., 2018). Interestingly, food restriction can induce CREB phosphorylation (Haberny et al., 2004; Ouyang et al., 2017), explaining the elevated pCREB levels observed in both CTR and DER animals undergoing FA training; this increase in pCREB levels could also underlie the observed food restriction dependent reward drive augmentation. Moreover, NAc activity has been correlated with increased social ranking (Shan et al., 2022). Specifically, the activation of D1 receptors in NAc seems to facilitate social dominance (Van Der Kooij et al., 2018). Consequently, social hierarchy instability caused by elevated general competition could be associated with excessive NAc activation. Taken together, these reports provide a strong argument connecting DER NAc overactivation with increased social instability. Furthermore, a recent report in bioarchives suggested that synergistic mPFC-NAc circuit activation encodes behaviors correlated with increased competition such as the approach of an opponent during a competing task (Tae-Yong et al., 2023). Interestingly, DER rats also displayed elevated vPFC activation providing additional evidence to the suggested neural correlate of unstable social structure.

We determined the effects of the DER experience on the behavioral output during sexual interaction and group food anticipation in adulthood. The DER experience did not affect sexual motivation or sexual consummation in contrast to other early life adversity models. DER animals displayed erratic reward learning and elevated unfocused exploration. These effects were accompanied by social instability during food access and abnormal vPFC and NAc overactivation. We propose that the stress induced by food restriction together with hierarchical instability led to the context-reward association deficits of DER rats. These effects can reflect pathophysiological characteristics observed in human victims of early life stress. By using group behavioral tasks, we simulate more accurately typical human life where everyday challenges include strong social components. Finally, the FA training protocol used in this study proved to be a versatile tool that allowed us to explore many behavioral aspects while also being sufficiently simple and customizable.

## Data availability statement

The raw data supporting the conclusions of this article will be made available by the authors, without undue reservation.

## Ethics statement

The animal study was reviewed and approved by Ethics Committee of the Faculty of Nursing, NKUA (Authorization number 291, Athens, 2019) Papadiamantopoulou 123, 11527 Athens Greece.

## Author contributions

ER: designed and executed the experiments, analyzed the data, and wrote the manuscript. DF: analyzed the data and wrote the manuscript. AS: designed and executed the experiments, analyzed the data, and revised the manuscript. All authors contributed to the article and approved the submitted version.
